# Traditional and biomedical care pathways for mental well‐being in rural Nepal

**DOI:** 10.1186/s13033-020-00433-z

**Published:** 2021-01-07

**Authors:** Tony V. Pham, Rishav Koirala, Brandon A. Kohrt

**Affiliations:** 1 Duke Global Health Institute, 310 Trent Drive, Durham, NC 27710 United States; 2 Transcultural Psychosocial Organization (TPO) Nepal, Baluwatar, Kathmandu, 44616 Nepal; 3 University of Oslo, Problemveien 7, Oslo, 0315 Norway; 4 Brain and Neuroscience Center Nepal, Krishna Dhara Marg, Kathmandu, 44600 Nepal; 5 Department of Psychiatry and Behavioral Sciences, George Washington University School of Medicine and Health Sciences, 2120 L Street, NW, Suite 600, Washington, DC 20037 United States

**Keywords:** Spirituality, Mental health, Shamanism, Medical anthropology, Traditional medicine, Ethnopsychology

## Abstract

**Background:**

There is increasing access to mental health services in biomedical settings (e.g., primary care and specialty clinics) in low- and middle-income countries. Traditional healing continues to be widely available and used in these settings as well. Our goal was to explore how the general public, traditional healers, and biomedical clinicians perceive the different types of services and make decisions regarding using one or both types of care.

**Methods:**

We conducted in-depth interviews using a pilot tested semi-structured protocol around the subjects of belief, traditional healers, and seeking care. We conducted 124 interviews comprising 40 traditional healers, 79 general community members, and five physicians. We then performed qualitative analyses according to a grounded theoretical approach.

**Results:**

A majority of the participants endorsed belief in both supernatural and medical causes of illness and sought care exclusively from healers, medical practitioners, and/or both. Our findings also revealed several pathways and barriers to care that were contingent upon patient-, traditional healer-, and medical practitioner-specific attitudes. Notably, a subset of community members duplicated care across multiple, equally-qualified medical providers before seeing a traditional healer and vice versa. In view of this, the majority of our participants stressed the importance of an efficient, medically plural society. Though participants desired a more collaborative model, no consistent proposal emerged on how to bridge traditional and biomedical practices. Instead, participants offered suggestions which comprised three broad categories: (1) biomedical training of traditional healers, (2) two-way referrals between traditional and biomedical providers, and (3) open-dialogue to foster mutual understanding among traditional and biomedical providers.

**Conclusion:**

Participants offered several approaches to collaboration between medical providers and traditional healers, however if we compare it to the history of previous attempts, education and understanding between both fields may be the most viable option in low- and middle-income contexts such as Nepal. Further research should expand and investigate opportunities for collaborative learning and/or care across not only Nepal, but other countries with a history of traditional and complimentary medicine.

## Background

“I anticipate that by the time you have children or grandchildren the art of *dhami*-*jhankri* (traditional healers) will be completely lost.” (female homeworker, age 71).

In low- and middle-income countries (LMIC), up to 40% of the population utilize traditional and complimentary medicine [1]. This is comparable to other settings such as sub-Saharan Africa [2] and high- and middle-income countries (HIC) where around half of the population also depends on traditional and complimentary medicine [3, 4]. Their appeal, whether a product of community standing [5] or psychosocial intervention [6, 7], offers a partial, but compelling answer to the mental health treatment gap in LMICs, wherein western treatments and explanations can run counter to local notions of wellness [8, 9] and 75% to 90% of the mentally ill forego treatment [10, 11]. Thus, the 2013–2020 World Health Organization (WHO) Mental Health Action Plan recommended government health programs include traditional and faith healers as treatment resources [12]. Since then, non-governmental organization (NGO) and government services have deployed traditional healers as non-specialists in manualized, low-intensity therapies or referral agents to such [13–15], otherwise known as task-shifting [16].

Against the backdrop of widespread use of traditional healing in LMICs, there is growing availability of mental health services in biomedical health services (e.g., primary care and specialty clinics). Over the past decade there has also been large scale proliferation of national mental health plans such as the Mental Health Gap Action Programme (mhGAP), a WHO plan to train and assess non-specialist providers and lay individuals to provide Western psychological interventions in more than 90 LMICs [17]. This raises the question of how traditional healers are involved in these efforts and what the future will hold for traditional healing in the treatment of mental illness. While task shifting presents an exciting opportunity to reduce the mental health treatment gap, folding indigenous healing methods into biomedicine presupposes the latter’s universal benefit over the former. For instance, consider how many evidence-based mental health treatments are based on data from populations which are predominantly western and biomedically educated [18]. Based on this premise, one could argue that conventional psychotherapy is no less an ethnocentric artifact than traditional healing. This may also explain why mental health interventions provided by non-local providers can lead to negative outcomes, wrong diagnoses, poor rapport, and reluctant followup [19–21], or why indiscriminate application of even culturally adapted psychotherapy can awaken or exacerbate distress [14, 22] or magnify conflict between dual systems of care [23–26].

Understanding the relationship between biomedical and traditional healing requires research on the intersection of these practices at a community level. Recent research in continents such as Africa and Asia has illuminated varying reasons for the traditional healer’s ongoing appeal over biomedicine [27]. For example, research has distinguished traditional healing in terms of its relative accessibility [28], exceptional prowess at treating certain quality of life issues [29], and overlap with sociopolitical, cultural, and historical context [30]. In light of these findings, we must redefine our notions of cultural inclusion if we aim to fully capture the traditional healer’s benefits on mental well-being, especially within settings such as LMICs where communities have long depended on indigenous healing practices [14, 22].

Before we delve into the traditional healer’s significance, we must first define the concept of a traditional healer. Per Nortje et al. (2016), traditional healers are “healers who explicitly appeal to spiritual, magical, or religious explanations for disease and distress” [6]. Based on this definition, a traditional healer would not include practices where we have seen a gradual shift from a classical towards a more biomedical conception of disease causation and treatment (e.g. Ayurveda, Traditional Chinese Medicine) [31, 32]. Several articles have reviewed the literature using this concept of the traditional healer. For example, one commentary reviewed the varying approaches to collaboration between traditional healers and medical providers. They found an overall lack of large-scale collaboration within LMICs when compared to HICs [27]. Further on this subject, one systematic review focused in on the state of collaboration between traditional healers and medical providers within LMICs. Their search yielded 13 publications from Africa and only one from Asia [33]. Despite widespread use of traditional healing systems such as Ayurveda and Traditional Chinese Medicine within Asia’s biomedical system [1, 6, 7, 34], have we neglected to incorporate the potential benefits of the more classical traditional healer?

Nepal exemplifies an Asian setting with proliferation of biomedical mental health services [35, 36] and a continued robust traditional healing landscape. Unfortunately, a vast majority of the research on traditional healing transpired between the 1970s and ‘80 s, the same time as when Nepal first opened its borders to foreign research [34]. To address the current gap in knowledge about how members of the public view these two systems and navigate them for care, we made it our research objective to better understand (1) preferences for treatment systems and (2) how future health policies can optimize community benefit of these systems with potentially parallel, complimentary, and conflicting approaches.

## Methods

### Overview

In quickly developing regions such southeastern Nepal, biomedical approaches have reportedly outpaced many traditional conventions [37–39]. In this study, we reexamine and recontextualize this view from a public health perspective using semi-structured in-depth interview protocols and current theories on qualitative data analysis. We investigate what factors drive patients to see traditional healers, what, if any, impact this may have on peripheral biomedical systems, and ultimately whether traditional healers can scale up mental healthcare within modern day Nepal. We present our study design, analysis, and findings according to the Consolidated Criteria for Reporting Qualitative Research (COREQ) [40].

### Setting

Nepal highlights the challenges of understanding the intersection of traditional and biomedical mental health services, raising the question of what the road ahead will be for people in the community who utilize these services. Nepal is considered one of the poorest nations in Asia and is classified with a “high warning” on Fund for Peace’s Fragile States Index [41]. In the face of various structural and socioeconomic barriers in Nepal, to date local non-governmental organization (NGO), governmental, and public health efforts have struggled to bridge the treatment gap in Nepal’s mental healthcare system. In the first barrier, 83% of Nepal’s rural residents live in poverty [42], and second, those in need of healthcare lack any insurance and must bear the entirety of their medical expenses [43]. As a result, ill and impoverished patients must not only pay for but also make sense of their illness before triaging its relative worth in time and money. For individuals who view mental illness in non-biomedical or stigmatized terms, many will never seek treatment [44].

In the aftermath of the 2015 earthquake of 7.8 magnitude, many high profile mental health projects emerged within rural Nepal only to witness a discrepancy between supply and demand [36]. For example, in 2008 the WHO initiated mhGAP in Nepal [45], and between 2012 and 2019, the UK Aid Direct and Department for International Development sponsored the Programme for Improving Mental Healthcare (PRIME), a project which scaled up mental health services by integrating it with primary care. Unfortunately, integration proved difficult and later analyses of both mhGAP and PRIME revealed that local understandings of mental illness and high levels of stigma precluded community awareness, demand, and access to basic mental health services [46, 47].

These factors together have made Nepal a cultural hotspot for investigating and chronicling indigenous healing methods which may have long serviced mental we[ll-being across Nepal. However, as with the broader research across Asia, Africa, and the rest of the world, most research took place over two decades ago [34]. Since then qualitative standards have improved their scientific openness, rigor, and reproducibility [48, 49] given increasing scrutiny [50–53]. Thus if we are to evolve our current literature base, we must first reinvestigate its foundations using today’s methodologies.

Our project focused on three rural village development committees (VDC) within one province of southeastern Nepal. For reasons of agreed anonymity, we withhold the specific names of our chosen worksites from publication. Nationally, we partnered with a local NGO, Transcultural Psychosocial Organization (TPO) Nepal. Locally, we collaborated with TPO Nepal’s satellite offices, village elders, and leaders who provided cultural advice and interpretation, facilitated and identified key-informants, and encouraged trust and respect between the research team and our chosen communities.

### Data collection

Between August and October 2019, we lived alongside and selected participants using purposive and snowball sampling. In comparison to more rigid sampling patterns, we relied on village elder advice and iterative participant referrals to better capture patients who would represent the region’s wide demographic variety. We conducted semi-structured in-depth interviews within a pre-selected location and performed all interviews within an area which respected privacy and the lack of environmental intrusion. We primarily relied on a Nepali interpreter (DL) and a mental health glossary compiled by TPO Nepal to ensure systematic translation between English and Nepali (https://bit.ly/2IQgY2X). RK, a Nepal-based psychiatrist, sat in periodically to offer feedback on translation. We kept interviews to an hour out of respect for the working lives of our participants, many of whom worked throughout the day as farmers or laborers. Per the suggestion of TPO Nepal, we compensated each participant a small amount of cash and household goods. TPO Nepal estimated this amount based off their strong history working in the field. They rationalized that a small amount of cash and household goods would serve as a sign of respect without coercing individuals into participating.

We also offered all participants a consent form before participating and included only those who consented in both verbal and written form. Although we did not catalog exactly which and how many participants declined to participate, we did note a few general observations. First, only a handful of participants from each village declined to participate, most frequently because they expressed being pre-occupied. In light of the negative media surrounding traditional healers, one community member and one drug retailer (“medical”) declined to interview for fear that we would cast them in a negative light. Two others declined to interview after expressing their lack of expertise on our research topics. One community member agreed to interview but declined to be audio recorded. All physicians agreed to interview but declined to be audio recorded.

TVP (male, American, Vietnamese ethnicity, MD psychiatrist, MscGH researcher) conducted semi-structured interviews using a pilot tested protocol which focused on the traditional healing landscape, patient illness narratives, and pathways to care [54]. During field work, participants came to view TVP primarily as a foreign researcher. We relied on our pilot tested protocol as reflection upon other tools, even those previously adapted for Nepal, revealed several areas for incompatibility. For instance, Craig and colleagues (2010) piloted the McGill Illness Narrative Inventory (MINI) within the Mustang district of Nepal and noted, “interviews that elicited the most nuanced narratives were the ones in which interviewers did the least amount of talking, and in which specific questions were put into dialogue with the interviewees’ responses to initial prompts in each section of the template, rather than those interviews that proceeded in a more methodical, checklist-like fashion” [55]. Similarly, we found the Barriers and Access to Care (BACE) scale somewhat rigid and lacking in terms of the tacit community biases relevant to our study [56]. Nevertheless, our finalized, semi-structured protocol drew inspiration from key, pertinent questions from both the MINI and BACE.

To promote conversational fluidity, TVP employed the piloted protocol less as a structured guide and more as a repository of questions. For example, after orienting the participant to our study, he allowed them to guide the interview as they saw fit. During moments which allowed for redirection, TVP focused on a select number of broad, key questions to anchor the participant within the scope of our study. He improvised follow-up questions based on context and the collection of questions pre-piloted by the interview protocol. A few representative questions included, “If you went to see a helper or healer of any kind, tell us about your visit and what happened afterwards;” “According to you, what caused your [patient’s own term for illness]?;” “What made that treatment work well?;” “Did you face difficulties affording care under the healer/medical practitioner?;” and “Did you face difficulties traveling to see the healer/medical provider?” We permitted participants to converse widely about not only themselves but others as well. We interviewed all participants at least once but more often several times. This helped us to develop rapport and increase the richness and time points of participant responses.

We completed same-day transcriptions and stored documents via encrypted cloud storage. To ensure the accuracy of our translations, RK cross-checked each of the audio files and transcripts for translational accuracy. We later performed qualitative analyses according to a grounded theoretical approach under the Straussian school of thought [57]. Two coders (TVP and DL) independently and manually reviewed, coded, and memoed each interview. As they coded the data they then added newly emerging codes to an iterative codebook. They completed the codebook upon reaching theoretical saturation, that is the point when no new codes emerged. Afterwards, TVP and DL outputted their coded data, and we as a team compared and contrasted their analyses while ensuring consistency between their analyses and the original data. We collaborated on how emerging codes fit within larger categories and themes and generated thematic concept maps. Overall, employing TVP and DL from data collection to analysis helped to ensure consistency from beginning to end. We cross-checked our manual analyses against in-depth computer-assisted qualitative data analysis (QualCoder Version 1.9) and key village leaders [58].

## Results

### Demographics

We conducted 124 in-depth interviews comprising 40 traditional healers, 79 other community members, and five physicians (Table 1). Traditional healer types included 33 *dhami*-*jhankri* (amalgam term for two separate traditional healer types), one *lama* (Buddhist monk and healer), and six *mata* (female healer). The mean age of traditional healers was 62 years (SD 13.0). 33 healers (n = 33; 82.5%) were male gender, 36 identified as Hindu (90.0%), 34 (85.0%) had no formal education, and 34 (47.5%) were from high caste Brahman groups. Among the 79 community-members, the mean age was 42.6 years (SD 20.2). 63 community members (79.8%) were female gender; 69 (87.3%) identified as Hindu; 61 (64.6%) had no formal education; and 38 (48.1%) were from high caste Brahman (priest caste) groups.

**Table 1:** Demographic Characteristics of 124 Participants Interviewed in Three Southeastern Village Development Committees of Nepal Between August and October 2019

	General Community (n = 79)	Traditional Healers (n = 40)	Physicians (n = 5)	Total (n = 124)
Mean age year (SD)	42.6 (15.8)	61.8 (13.0)	36.8 (2.9)	48.6 (17.2)
Gender (% male)	20.2	26.6	100.0	50.8
Religion (%)				
Hindu	69 (87.3)	34 (85.0)	5 (100.0)	108 (87.1)
Buddhist	7 (8.9)	6 (15.0)	0	13 (10.5)
Christian	3 (3.8)	0	0	3 (2.4)
Caste (%)				
Low castes	10 (8.1)	8 (20.0)	0	18 (14.5)
High castes	38 (48.1)	19 (47.5)	5 (100.)	62 (50.0)
Newar	0	1 (2.5)	0	1 (0.8)
Kirati	9 (11.4)	4 (10.0)	0	13 (13.1)
Indigenous Middle Hills	4 (3.2)	3 (7.5)	0	0
Indigenous Tarai	17 (21.5)	5 (12.5)	0	0
Birthplace (%)				
Eastern Middle Hills	23 (29.1)	17 (42.5)	0	40 (32.3)
Eastern Tarai	47 (59.5)	18 (45.0)	2 (40.0)	67 (54.0)
Eastern Himalaya	1 (1.3)	1 (2.5)	0	2 (1.6)
Central Middle Hills	0	1 (2.5)	0	1 (0.8)
Kathmandu	2 (2.5)	0	3 (60.0)	5 (4.0)
Bhutan	1 (1.3)	0	0	1 (0.8)
Hong Kong	0	1 (2.5)	0	0
India	4 (3.2)	0	0	0
Traditional healer category (%)				
Dhami-jhankri	0	33 (82.5)	0	33 (26.6)
Lama	0	1 (2.5)	0	1 (0.8)
Mata	0	6 (15.0)	0	6 (4.8)
Occupation (%)				
Farmer	21 (26.6)	19 (47.5)	0	40 (32.3)
Blacksmith	0	1 (2.5)	0	1 (0.8)
Driver	1 (1.3)	0	0	1 (0.8)
Housewife	41 (51.9)	4 (3.2)	0	44 (3.2)
Female community health volunteer	1 (1.3)	0	0	1 (0.8)
Caretaker	1 (1.3)	0	0	1 (0.8)
Military	1 (1.3)	0	0	1 (0.8)
Mechanic	2 (2.5)	0	0	2 (2.0)
None	2 (2.5)	0	0	2 (2.0)
Physician	0	0	5 (100.0)	5 (4.0)
Education (%)				
None	51 (64.6)	34 (85.0)	0	85 (65.9)
Some Primary School	15 (19.0)	5 (12.5)	0	20 (16.1)
Primary School	8 (10.1)	1 (2.5)	0	9 (7.3)
Bachelors	5 (6.3)	0	0	5 (4.0)
Masters and Above	0	0	5 (100.0)	5 (4.0)

### Overview of care pathways

**Fig. 1:**
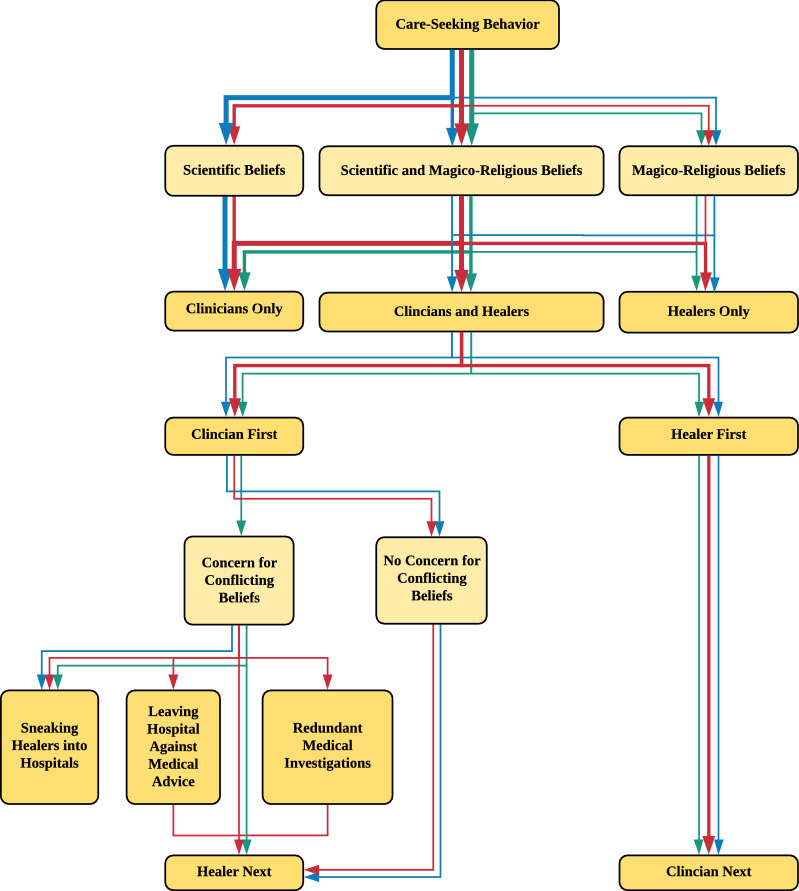
Patient Pathways, Perceptions, and Barriers to Care Among Healers and Clinicians. Blue = Physician Endorsement; Green = Healer Endorsement; Red = Other Community Member Endorsement; Small Arrow = Less than 25% Endorsement; Medium Arrow = 25% to 45% Endorsement. Large Arrow = Greater than 45% Endorsement; Light Yellow = Participant Attitude; Dark Yellow = Participant Action]

Based on the qualitative analysis, we generated a visualization on care pathways (Fig. 1). Each component of the care pathway is described below. The frequencies of themes and sub-themes by respondent type are provided in Table 2. For brevity and comprehensibility, we refer to traditional healers as “healers,” medical professionals of any background as “clinicians,” and patients of either healers or clinicians as “patients” [54].

**Table 2:** Frequencies and percentages of themes across all participant types

	Endorsing Participants			
	Traditional Healer (%)	Community Member (%)	Physician (%)	Total (%)
Personal explanatory models for disease				
1. Supernatural and scientific beliefs	36 (90)	40 (50.6)	2 (40.0)	78 (62.9)
1a. Self-contradicting belief narratives	7 (17.5)	21 (26.6)	0	28 (22.6)
2. Science alone	0	26 (32.9)	5 (100.0)	31 (13.7)
3. Supernatural alone	1 (2.5)	2 (1.6)	1 (20.0)	4 (3.2)
Perceptions of traditional healers				
1. Origin stories for supernatural relationships and abilities	21 (52.5)	15 (19.0)	0	36 (29.0)
1a. No fear or difficulty versus mental illness	1 (2.5)	6 (7.6)	0	7 (5.7)
2. Healer-induced feelings of peace and satisfaction	6 (15.0)	21 (26.6)	2 (40.0)	29 (23.4)
2a. Resultant desire to re-enter everyday life	5 (12.5)	12 (15.2)	2 (40.0)	18 (14.5)
3. Patients prefer one healer based on healer-factors	3 (7.5)	16 (20.3)	0	19 (15.3)
3a. Healer as community member	21 (52.5)	52 (65.8)	0	73 (58.9)
3b. Healer as figurative or literal family	4 (10.0)	12 (15.2)	0	16 (12.9)
3c. Sensitivity to local socioeconomic issues	10 (25.0)	45 (57.0)	0	55 (44.4)
3d. Non-discriminatory	20(50.0)	33 (41.8)	0	53 (42.7)
3e. Offers skills as a social service	14 (35.0)	36 (45.6)	0	50 (40.0)
3e1. Realistic and familiar payment scheme	16 (15.0)	29 (72.5)	0	35 (28.2)
3e2. Avoids growing wave of healer corruption	13 (32.5)	26 (29.1)	3 (60.0)	36 (29.0)
3e3. Emotional availability	1 (2.5)	11 (13.9)	0	12 (9.7)
3e4. Genuine appearance	0	51 (64.6)	8 (20.0)	59 (47.6)
3e4a. No substance abuse	0	6 (7.6)	0	6 (4.8)
3f. Quick response	3 (7.5)	18 (22.8)	0	21 (16.9)
3g. Follow-up care	6 (15.0)	12 (15.2)	0	18 (14.5)
3h. Allows for reinvestigation across several healers	0	15 (19.0)	0	15 (12.1)
3h. Proven mastery over the supernatural	13 (32.5)	29 (36.7)	0	42 (33.9)
3h1. Has wide repertoire of mantra	1 (2.5)	5 (6.3)	0	6 (4.8)
3i. Can instill some but not too much fear	1 (2.5)	13 (16.5)	0	14 (11.3)
3j. Lacks arrogance	12 (30.0)	41 (51.9)	0	53 (42.7)
Perceptions of clinicians				
1. Well-educated	24 (60.0)	64 (81.0)	1 (20.0)	89 (71.8)
2. Dedicated to medicine	9 (22.5)	32 (40.5)	2 (40.0)	43 (34.7)
3. Adverse prescription side effects	19 (47.5)	24 (30.4)	0	43 (34.7)
4. Powerful and fast-acting medicines	11 (27.5)	56 (70.9)	0	67 (54.0)
5. Financial barriers	12 (30.0)	32 (40.5)	0	44 (35.5)
6. Geographic barriers	7 (17.5)	29 (36.7)	0	36 (29.0)
7. Patient preference against discriminatory practices	5 (12.5)	8 (10.1)	0	14 (11.3)
Perceptions of care among healers and clinicians				
1. Healers alone for specific ailments	4 (10.0)	33 (41.8)	1 (20.0)	38 (30.6)
1a. Subjective symptomatic improvement	0	19 (24.1)	0	19 (15.3)
1b. Minor physical illnesses	8 (20.0)	21 (26.6)	0	29 (23.4)
1c. Intractable physical illnesses	0	12 (15.2)	0	12 (9.7)
1d. Odd behavior	7 (17.5)	15 (19.0)	0	22 (17.7)
1e. Madness	9 (22.5)	5 (6.3)	0	14 (11.3)
1f. Socio-environmental duress	5 (12.5)	14 (17.7)	0	19 (15.3)
1fa. Taboo subjects	2 (5.0)	11 (13.9)	0	13 (10.5)
2. Clinicians alone for specific ailments	11 (27.5)	39 (49.4)	3 (60.0)	53 (42.7)
2a. Severe or emergency physical illnesses	20 (50.0)	26 (32.9)	0	46 (37.1)
2b. “True” mental illness	11 (27.5)	4 (5.1)	0	15 (12.1)
3. Both healers and clinicians for specific ailments	12 (30.0)	51 (64.6)	1 (20.0)	64 (51.6)
3a. Healers as first-line	9 (22.5)	35 (44.3)	1 (20.0)	45 (36.3)
3a1. Rule out supernatural issues	4 (10.0)	17 (21.5)	0	21 (16.9)
3a2. Rule out must precede clinician’s	1 (2.5)	11 (13.9)	0	12 (9.7)
3b. Clinician as first-line	5 (12.5)	35 (44.3)	1 (20.0)	41 (33.1)
3b1. Concern for conflicting belief systems	4 (10.0)	28 (35.4)	0	32 (25.8)
3b1a. Resultant unfulfilled expectations	1 (2.5)	22 (27.8)	1 (20.0)	24 (19.4)
3b2. No concern for conflicting belief systems	0	8 (10.1)	1 (20.0)	9 (7.3)
3b2a. Resultant unfulfilled expectations	0	2 (2.4)	0	1 (2.4)
3b3. Unconventional medical care pathways	1 (2.5)	13 (16.5)	1(20.0)	15 (12.1)
3b3a. Multiple, redundant clinicians	0	9 (11.4)	0	9 (7.3)
3b3b. Bringing healers into the hospital	0	5 (6.3)	1 (20.0)	6 (4.8)
3b3c. Early discharge to healer	1(2.5)	4 (5.1)	0	4 (3.2)
Desired pathways to care among healers and clinicians				
1. Care-seeking as it stands	1 (2.5)	19 (24.1)	4 (80.0)	23 (18.6)
1a. Healers and clinicians too fundamentally different	1 (2.5)	9 (11.4)	1 (20.0)	10 (8.1)
1b. Concern for harm from magical-medical mixing	3 (7.5)	10 (12.7)	0	13 (10.5)
2. Improved collaboration	27 (67.5)	45 (57.0)	0	72 (58.1)
2a. Legitimizes the healer’s role in society	2 (5.0)	4 (5.1)	0	6 (4.8)
2b. Preserves Nepali culture	3 (7.5)	3 (3.8)	0	6 (4.80)
2c. Medical training and referrals to clinicians	2 (5.0)	4 (5.1)	0	6 (4.8)
2d. Two-way referrals	6 (15.0)	9 (11.4)	0	16 (12.9)
2e. Mutual respect and recognition	17 (42.5)	9 (11.4)	0	26 (21.0)
2e1. Learning and motivating one another	5 (12.5)	2 (2.5)	0	7 (5.7)
2e2. Sharing skill sets	2 (5.0)	1 (1.3)	0	3 (2.4)

### Explanatory Models for Mental Illness: Scientific and Magico-religious beliefs

Community members who presented to healers focused on everyday chronic-remitting frustrations with either medical or magico-religious explanatory frameworks (magico religious referring to “beliefs prevalent in a particular culture concerning various supernatural influences operating in the environment” [59]. Community member complaints could bely a complex depth of emotional, socioeconomic, and environmental duress, however, few voiced their frustrations in direct relation to mental illness. Rather, community members focused on sickness, unfulfilled medical expectations, and supernatural discord using terms such as *paagal* (generic, stigmatizing term for madness and related constructs), problems or difficulties in the *man* (‘heart-mind’, a non-strigmatizing term which can loosely refer to emotions and memory processes conceptualized to emanate from the location around the heart), problems or difficulties in the *dimaag* (‘brain-mind’, term for cognitive and social behavioral control processes associated with the physical brain; considered a stigmatizing term when referring to a brain-mind disorder, e.g., *dimaag bigrieko*, ‘broken brain-mind’), and problems related to the *saato* or *atma* (non-stigmatizing terms for the ‘soul’ tied to various emotional and physical states) [55]. However, the opportunities to employ less stigmatizing phrasing, e.g. in terms of the *man* or *saato/atma* tended to arise in relation to healers moreso than clinicians (see Additional file 1: S1, Quote #1). The majority of participants adhered to both medical and magico-religious care seeking behavior (n = 78; 63%). Few respondents reported adhering to only medical beliefs (n = 31; 14%) or only to magico-religious beliefs (n = 4; 3%) (see Additional file 1: S1, Quote #2).

Nearly a quarter of participants (n = 28; 23%) initially reported that they only adhered to biomedical treatment seeking but then disclosed also employing or observing effective magico-religious healing. For example, one 54-year-old farmer initially likened mental illness to a *phuteko shisha* (broken glass) which a healer could not repair. Nevertheless, he later described a healer who cured a young man’s mental deterioration from academic turmoil. Multiple community members described a young farmer who lost consciousness while cutting grass (n = 9; 7%). The young man’s wife implored him to see the local *mata* (female healer) for *lagne* (supernatural affliction). He complied, albeit reluctantly, with the *mata*’*s* two-day *phukne* (blowing technique). Afterwards, he promptly recovered and returned to work with a newfound belief in healers.

### Perceptions of healers

Rather than choosing healers based upon their caste, religion, or attitudes towards medicine, community members described healer-specific factors which could mediate faith (n = 19; 15%) including the healer’s status within the community, genealogical or symbolic familial ties, sensitivities to local socioeconomic issues, level of discrimination among the poor, emotional availability, level of genuine appearance, and mastery over the supernatural. As one 24-year old homemaker put it, “Without trust, connection, or belief you cannot visit the *dhami*-*jhankri*.”

Community members valued healers as active community members (n = 73; 59%), relatives, or providers who treated patients like family (n = 16; 13%). On this subject, one 66-year-old ex-military man shared his story, “We traveled 22 km to reach this *dhami*-*jhankri* (healer). I came to know about him through my wife’s family. My two brother-in-laws are his students. At first we went to my brother-in-laws [for treatment] but they were quite busy.”

The healer’s enmeshment within their community alerted them to patient circumstances which precluded payment (n = 55; 44%). As one 50-year-old farmer explained, “*eutai taalko maachale aphne taalko maachalai sahayog garna sakchha*” (only fishes from the same lake can help each other). Community members reported that nearby healers could treat within a moment’s notice (n = 21; 17%) and follow-up until symptomatic improvement was achieved (n = 18; 15%). As a 49-year-old farmer described, “I returned home following the *dhami*-*jhankri’*s treatment. Later on, he talked to me over the phone and asked, ‘Are you improving?’ I told him, ‘I feel better,’ and then asked that he treat me again, and so he did.”

Community members valued the healer’s accessibility for mild issues (e.g., stomach upset, headache, cough, allergies, pain) that would not merit a time-consuming visit to see a biomedical health worker at a local health post. Given their relative accessibility and abundance, some community members reinvestigated chronic-remitting issues across multiple healers (n = 15; 12%), sometimes to the patient’s detriment (see Additional file 1: S1, Quote #3). Community members also appreciated how most healers did not discriminate based on caste, ethnicity, or wealth (n = 53; 43%) and provided their services as a *samaajik kaam* (work of service) (n = 50; 40%). In the words of one 84-year-old farmer, “Whoever has *lagne* (supernatural affliction) will go [to the healer]. Touchable, untouchable, *damai*, *kami*, *pode*, *sarki*, *chhetri*, *bahun* (various lower castes) – whoever has *lagne* will go.”

Healers charged according to a sliding scale donation embedded within familiar payment schemes (n = 35; 28%). For example, patients could place *bheti* (an offering to the gods) on a *pujako thaali* (ceremonial rice plate) (See Additional file 1: S1, Quote #4). However, community members feared a growing wave of corruption and *fatai* (bad work) among healers fueled by money, power, and fame and avoided healers who discriminated against the poor (n = 36; 29%). They associated discrimination with apathetically deferring treatment or feigning excuses to treat (n = 12; 10%). From the perspective of a 28-year-old farmer, “Many *jhankri* (healers) here don’t prioritize poor families. They’ll take one look at our clothes or *bheti* (offerings to the gods) and discriminate against you, not openly, but through their *man* (heart-mind).” Several disliked healers with a penchant for excessive alcohol (n = 6; 5%). As a 38-year-old farmer added, “One *dhami*-*jhankri* appeared intoxicated when I met with him, telling me to do this or that. I didn’t believe anything he said and never used him again.”

On the contrary, community members preferred healers whose *man* (heart-mind) appeared genuine, dignified, and kindhearted (n = 59; 48%). Beyond factors related to the healer’s character, community members preferred healers who possessed a skill level commensurate with their needs. To gauge a healer’s capacity, many drew from reported feats which showcased the healer’s supernatural prowess (n = 42; 34%). Anecdotes of their feats often took the form of origin stories which contextualized their uncanny strength, clairvoyance, and casual acquaintances with spirits, ancestors, and gods (n = 36; 29%). The healer’s supernatural relationships and abilities specifically allowed for fearless and effortless treatment of odd behavior and *paagal* (madness) (n = 7; 6%). While community members could view fearful healers with reverence, others viewed excessive fear-making as off-putting (n = 14; 11%) (see Additional file 1: S1, Quote #5).

Community members paid particular attention to a healer’s ability to mentally focus and chant *mantra* (enchantments), a skill necessary to commune with supernatural entities, deities, and holy scripture such as the *Vedhas* (Sanskrit religious text). Consequently, community members desired for healers to possess a wide repertoire of *mantra* (n = 6; 5%). Others preferred healers who sub-specialized in specific ailments, whether biomedical or supernatural, with at times a hap-hazard approach to gauging who specialized in what (see Additional file 1: S1, Quote #6).

Beyond physical recovery, efficacious treatments could induce particular states which community members described using local notions of wellness, e.g. *atma santusti* (satisfaction in the soul), *manko shanti* (peace in the heart-mind), *manko kura* (thought of mind), *halka man* (lightness of the heart-mind) and so forth (n = 29; 23%). Achieving peace and satisfaction meant they could re-enter everyday life (n = 18; 15%). From the perspective of a 33-year-old homemaker, “We use the same healer every time, and he has been like a personal *dhami* (healer) for us. No matter what time it is our *dhami* will come and perform his treatment.”

Although community members preferred proven healers, they simultaneously rejected healers who appeared *thulo bhayo* (those who had become big or arrogant) (n = 53; 43%), as evidenced by exclusively believing in their own skill-set, overextending their skills into dispensing medicine, excessively competing with other providers, whether healers or clinicians, displaying their work or powers without solicitation, or requesting unrealistic sums of payment. One healer described how the pressures of this expectation compelled him to treat in all circumstances (see Additional file 1: S1, Quote #7).

These opinions contrasted with those from Christian respondents who explicitly denied belief in the healer’s interventions. To explain this attitude, one Hindu shopkeeper noted, “There have been many [Christian] conversions. They (the converted) criticize us (non-Christians) for having backward beliefs [in healers]. Still, we cannot forget our culture or tradition and what we have followed since our ancestor’s time.” Ultimately, the dynamic between Christian and Hindu beliefs may offer more nuance than is readily apparent. For example, one middle-aged Hindu woman told us about her Christian uncle who reluctantly received treatment from a healer, only to witness his own recovery (see Additional file 1: S1, Quote #8).

### Perceptions of clinicians

According to community members, clinicians dedicated themselves to medicine (n = 43; 35%). As a result, they possessed a strong educational background (n = 89; 72%) that allowed them to astutely diagnose and treat biomedical illnesses using advanced technologies afforded only through modern science. Among their technologies, community members especially held their prescription medicines and “injections” (catch-all-term for shots or intravenously administered treatment) in high regard. Prescription medicines and injections, though prone to adverse side effects (n = 43; 35%), could act both powerfully and fast (n = 67; 54%). Given the recent developments in roads and communication, community members reported easy access to medical amenities. However, poverty (n = 44; 35%) and life within remote villages (n = 36; 29%) could preclude access.

Community members appreciated clinicians for their hard work and dedication to the community. Community members especially appreciated clinicians who could *ramrari bolne* (speak nicely) and understand the patient’s socioeconomic conditions. However, others reported a small trend among clinicians who withheld premium services from the poor (n = 14; 11%). One 32-year-old farmer stated, “There are some doctors who get irritated and act rude towards their patients. They will take one glance at their patients, judge them, and then treat them on this basis.” Overall though, community members viewed clinicians as a critical advancement in the health and overall wellness of their community.

### Perceptions of care among healers and clinicians

Community members who exclusively endorsed seeking biomedical care (n = 53; 43%) reported seeking their help for *dherai aakashmic rog* (very sudden medical illnesses, e.g., medical emergencies) (n = 46; 37%) and *sakali manasik rog* (true mental illnesses) (n = 15; 12%). On the other hand, community members who reported only using traditional services reported eventual, though usually temporary, improvement from minor (n = 29; 23%) or intractable physical illness (n = 12; 10%), odd behavior (n = 22; 28%), *paagal* (madness) (n = 14; 11%), and socio-environmental distress (n = 19; 15%).

Most socio-environmental issues circled around taboo subjects such as domestic violence or suicide (n = 13; 10%). One family pleaded to a nearby healer because of nightly, inexplicable sounds coming from the kitchen, namely of clanging plates, opening cabinets, and blowing wind. The healer discussed their complaints in reference to the mother who had recently hanged herself. The family with the healer performed *chinta* (a night long healing ceremony), applied kerosene across seven pieces of bamboo, a culturally relevant item when carrying the dead, and burned the tree from which the mother hanged herself. Since then, no sounds bothered the family again.

A majority of the community members used both biomedical and traditional services for illnesses with either magico-religious or biomedical explanation (n = 64; 52%) (see Additional file 1: S1, Quote #9). Many community members sought traditional services first because of various reasons (n = 45; 36%). In addition to the economic and access barriers to modern medicine, community members reported fear of “injection” and “saline” treatments adversely interacting with magico-religious illness (n = 21; 17%). For example, a clinician’s treatment could lose its efficacy if not preceded by a healer’s (n = 12; 10%) (see Additional file 1: S1, Quote #10). Worse than inefficacy, adverse reactions could also disable or even kill a patient. As one 39-year-old farmer noted, “I had a relative who went to the hospital for *lagne* (supernatural affliction). Because my relative did not see the *jhankri* (healer) first, the medical treatment turned him into an *aapanga* (person with a leg disability).” Another 43-year-old shopkeeper described a similar scenario in which a hospital patient who should have sought the healer’s treatment first became *langado* (person with a leg amputation).

On the other hand, community members who first pursued biomedical care reported not fearing adverse medical-magico-religious reactions (n = 41; 33%). Instead, these community members feared clinicians who would scrutinize their magico-religious concerns (n = 32; 26%). For instance, one group described clinicians who might scornfully say, “if you went to the healer then why did you bother coming here?” To which one discussant remarked, “That’s why we never say anything about *dhami*-*jhankri* in front of doctors.” (n = 3; 2%) (See Additional file 1: S1, Quote #11).

Conversely, several community members described *ustai sochne* (like-minded) clinicians who disarmed them in light of their shared supernatural concerns (n = 9; 7%). This contrast between like-minded and non-like-minded providers was notable as patients who perceived ridicule about their *andha biswas* (backwards beliefs) or *purana dharana* (traditional mindsets) also reported medical dissatisfaction (n = 24; 19.4%) more frequently than those who didn’t (n = 2; 2%). Patients complained about (1) intervention type, power, or speed not in line with their expectations, (2) diagnostic failure, or (3) poor astrological alignment with their clinician.

Among the medically-dissatisfied patients, many went on to craft elaborate treatment sequences without informing clinicians who they feared would scrutinize them for adhering to a traditional mindset (n = 15; 12%). A group of female shop-goers discussed their thoughts on the matter. The 32-year-old shopkeeper prefaced, “People put their faith in *dhami*-*jhankri* when the doctors have yet to cure them.” Her 22-year-old friend further illustrated, “Let’s say someone had a fever for two or 3 days. That person may get better but they will still feel that doubt in their *man* (heart-mind). So they’d see the *dhami*-*jhankri*.” Another 40-year-old woman added, “We go to doctors first because they give us medicines. If we don’t get better after taking their medications then we go to see the *dhami*-*jhankri*.” Finally, a 36-year-old woman concluded, “We go to whomever we trust the most. We go in order to console ourselves. That’s why after going to the doctor we have to go back to the *dhami*-*jhankri* whether or not the doctor knows.”

In another example, some dissatisfied outpatients did not comply with medications and duplicated medical care across disconnected, equally-qualified clinicians (n = 9; 7%). These patients expressed particular dissatisfaction with their medications working much slower than their initial expectations and thus gave up before completing the clinician’s prescribed duration. When they doubted one clinician, rather than contacting that clinician again, they instead sought the care of another clinician without informing them about the prior provider. The next clinician could then offer the same level of care and results as the prior (see Additional file 1: S1, Quote #12). Patients could repeat this process seemingly without end until symptomatic resolution. One community member reportedly saw seven clinicians for the same medical complaint until symptomatic resolution.

Several community members reported furtively bringing healers into the hospital, most frequently for protection against malevolent spirits who died in the hospital (n = 6; 5%). Other inpatients requested early discharge to see a healer; this was typically done against medical advice (n = 4; 3%) (See Additional file 1: S1, Quote #13). When we informed clinicians about these various trajectories many expressed surprise or disbelief. For instance, the vice director from one nearby hospital doubted the healer’s very existence in the surrounding areas. Some biomedical clinicians were aware of these different care-seeking pathways. A nearby medical director and cardiologist classified care-seeking within the community in one of three ways: 1) healers only, 2) healers before doctors, or 3) doctors before healers. He also acknowledged inpatients who would bring healers and doctors who looked the other way.

In one instance of provider plurality, a doctor referred a 31-year-old homemaker to a healer who blessed the doctor’s medical treatment. Another neurosurgeon recommended a 65-year-old homemaker who feared supernatural involvement to see a healer following his treatment. One psychiatrist permitted patients, most often with conversion disorder, one-time treatments from a healer. However, such collaborative efforts were few in number, and the medical director admitted that a majority of clinicians unintentionally siloed traditional, ostensibly non-compliant, patients into a lower stratum of medical priority, thus widening the medical treatment gap between rural and urban care.

### Desired pathways to care among healers and clinicians

We asked residents to reflect on their future desires for improved healthcare access and quality, whether traditional or biomedical. Several community members advocated for healer-clinician relationships to stay as they were (n = 23; 19%), with several citing fundamental opposition between the two schools of thought (n = 10; 8%). Other community members feared collaboration because of the potential for adverse interactions between biomedical and supernatural forces (n = 13; 10%).

On the other hand, most participants favored stronger collaboration between their local clinicians and healers, citing general benefit for the community (n = 72; 58%). As one 45-year-old shopkeeper described, “If doctors and *dhami*-*jhankri* work together then normal citizens like us will get better services.” Other community members cited specific reasons. For example, several community members anticipated that collaboration would sanction the healer’s presence in the community and hospital (n = 6, 5%). A few healers viewed collaboration as a means to save a critical aspect of Nepali culture from eventual extinction (n = 6; 5%). One 54-year-old healer even viewed the current model as inherently dangerous, “Doctors and *dhami*-*jhankri* should discuss healthcare in Nepal. Right now, some *dhami*-*jhankri* do too much. Their patients may die if they don’t receive proper medical treatment.” Although there was a lack of broad consensus about how collaboration could push forward, participants proposed three broad areas to focus upon.

In one proposal, several community members suggested that healers train in medical diagnosis and referrals to clinicians (n = 6; 5%). As one 28-year-old homemaker put it, “The *dhami* should be knowledgeable. They should be able to identify the cases that either *dhami* or doctors have to treat.” To this end, one woman passionately delivered a story that started with her family finding a bite mark on her niece. The family then summoned a nearby healer who attributed the bite mark to a stray mouse. Regardless, her niece passed away later that night (see Additional file 1: S1, Quote #14). A post-mortem revealed a snake bite. “They could have saved her life,” the woman thought, “had only the healer communicated with a doctor.” For further evidence in favor of biomedical training, other participants turned to past federal and hospital attempts at healer accreditation (see Additional file 1: S1, Quote #15). To date, however, efforts at biomedical training have reportedly lacked follow through (see Additional file 1: S1, Quote #16).

In contrast to a model which focuses on biomedical training and one-way referrals to clinicians, other participants endorsed an approach which favored two-way coordination between healers and clinicians (n = 16; 13%) (see Additional file 1: S1, Quote #17). For example, community members suggested that healers and clinicians could refer patients to one another based on their presenting complaint (see Additional file 1: S1, Quote #18). Two healers suggested communal spaces in which both healers and clinicians could treat patients (see Additional file 1: S1, Quote #19).

As for the most popular proposal, nearly a quarter of the participants suggested mutual respect, recognition, and understanding over formal biomedical training and referrals (n = 26, 21%). A model focused on education would encourage healers and clinicians to learn from each other (n = 7; 6%) and even share techniques (n = 3. 2%). As one healer emphasized, “Both doctors and *dhami*-*jhankri* should understand each other and discuss their treatments and patients. Only then will patients get better.” Having witnessed the lack of follow through from prior attempts at full-fledged collaboration, one 50-year-old healer viewed mutual understanding as a healthy middle ground towards the healer’s inclusion: “*euta manchele arule bhaneko aadhi matrai sunnu parcha ani aru aphai jana dinu parcha*” (one must listen to 50 percent of their (healer) sayings and let the rest be gone). The medical director similarly advocated for mutual understanding. While he expressed a desire for a cross-collaborative and robust referral system, he anticipated that such a model would prove impractical in the context of scarce governmental oversight.

## Discussion

In Nepal, a great body of literature has chronicled the healer’s role in treating mental well-being, mostly between the 1970s and ‘80 s [34]. In our study, we circle back to the present, notably within the southeastern regions of Nepal where growing biomedicalization of mental health care has reportedly subsumed the healer’s role. We sampled general community members, physicians, and healers using semi-structured interviews. We focused discussion on prevailing beliefs, pathways to care, and health models which would meet the needs of both community and biomedicine. Two overarching themes emerged across our qualitative analyses. First, within the southeastern regions of Nepal, people still see healers because of several provider factors that impacted quality of care, some of which spanned across traditional and biomedical practices. Second, general community members, healers, and clinicians altogether desired a more collaborative and structured model.

Broadly speaking, participants continued care among healers despite unprecedented growth in mental health services across Nepal. Several healer-specific factors could impact quality of care. For instance, whereas community members viewed clinicians as “fish from another pond,” community members viewed healers as neighbors who knew the local experience, whether familial or socioeconomic. As neighborhood healers, a patient could call upon their neighbor for assistance with any number of ailments, whether biomedical or magico-religious. Rather than allopathic medications and “injections,” community members associated healers with tradition, the supernatural, and Ayurvedic medications which acted slow and offered minimal unwanted side-effects. Rather than strict biomedical categories for distress and disease, a patient seeking healer-care could converse within local explanatory frameworks or magico-religious phenomena, e.g. *bhut pret* (ghost possession).

Reflection upon our pilot study study suggests that this magico-religious interface works through patient belief, social support, symbolic transference and narration, therapeutic alliance, healer empathy, expectations of recovery, and cultural models of distress [54]. These mechanisms, in keeping with literature throughout Nepal, can elicit a subjective rise in mental well-being sometimes expressed through local terms of personal and societal wellness, e.g. *atma santushti* (satisfaction in the soul) [34, 54]. Viewed through a biomedical lens, healers may treat in the same way placebo can alleviate pain [60], gastric tachyarrhythmia, motion-induced nausea [61], and so forth.

Our results suggest that community members who seek healers do not mutually exclude themselves from clinicians. In Nepal, clinicians, whose treatments have been made more accessible than ever due to modern road development, helped community members in ways that healers could not, be it through allopathic medications or so called “injections.” This parallels broader research from high-income countries in which patients turned to traditional and complementary medicines while expressing no less satisfaction with conventional medicine than those who preferred biomedicine exclusively [62–64].

The lack of mutual exclusivity between traditional healing and biomedicine may also reflect an area of overlap between the fields. For instance, cost could preclude access for both clinicians and healers. Even more surprising, many community members scarcely took stock of their providers specific belief system, preferring more that they, whether clinicians or healers, treat patients with an open mind. If community members perceived scrutiny between ostensibly paradoxical beliefs (e.g. traditional versus biomedicine) community members sometimes siloed their care or went through painstaking efforts to establish care with both (e.g. requesting for an early hospital discharge to see a healer, seeing a healer in the hospital without informing their medical team). Given the concomitant intersection between traditional and biomedical pathways, accounts of magico-religious recovery could coincide with a patient’s, arguably, unrealistic expectations of biomedical recovery.

In view of these factors and pathways, general community members, healers, and physicians came together in their support of broader collaboration, the second overarching theme from our study. Participants discussed a myriad of approaches, from which three primary ideas emerged: (1) training healers in biomedicine and referrals; (2) promoting formal, two-way collaboration between healers and clinicians; and more simply; (3) engendering mutual respect between the two disciplines. If we contextualize these ideas within Nepal’s history of research and public policy, it becomes evident that understanding and education about traditional healing provide the most feasible pathway towards culturally appropriate therapies (see Additional file 2: S2 for a detailed breakdown).

## Limitations

For full ethnographic richness, we planned month long stays within each village. In reality, finding accommodations within resource-poor villages proved difficult and though many opened their doors to us, we restricted ourselves accordingly. To select participants, we relied on purposive and snowball sampling to remove artificial pretense around our village presence and to foster naturalistic interviews within community subsections previously unknown to us, however, this in turn removed systematic control which would have been afforded by conventional sampling methods. We broadly sampled across three well-scattered rural VDCs to capture the southeastern region’s diversity. Unfortunately, the southeastern region of Nepal, which consists of six districts across two provinces, was in fact larger and more diverse than our sampled VDCs [65].

We attempted to include more systematized approaches with varying results. For example, we used a piloted semi-structured interview protocol to solicit illness narratives, however we improvised with rather than against patient redirection and facilitated interviews of varying length and subject-matter. To structure data analysis, we subscribed to a grounded theoretical approach, however this did not protect from subjectivity in retrospective report. Though we systematized translation by using a mental health glossary and guidance from a Nepali-trained psychiatrist, translation in itself proved difficult as many terms and concepts offered no clear translation between English and Nepali. Finally, we deduced, rather than induced, causality among the resultant themes to construct our pathways, perceptions, and barriers to care diagram, thereby introducing further bias into our analyses.

## Recommendations

Our results, along with those from a prior scoping review on healers and mental well-being in Nepal, suggest multiple areas for future research [34]. As it stands, our primary understanding of Nepali healers stems from ethnography which took took place over two decades ago. Since this time, many advancements in qualitative and quantitative research have emerged. Thus, the literature base as it stands merits reinvestigation and replication, especially among researchers independent from the original findings [52]. We must revisit the healer landscape not only within the southeastern regions of Nepal, but across Nepal’s other regions that offer significant cultural and geographic diversity. Beyond Nepal, other settings deserve research attention as well. Healers have served various populations across other LMICs [1, 6, 7, 27, 33, 66–68]. Asia in particular lacks a recent literature base commensurate with its size, geography, and history of traditional beliefs. Even within HICs, we see an emerging trend of traditional, complimentary, and alternative medicines [1, 27]. Given the interest in collaboration within our study, Nepal, and the world en masse, we propose three areas for mixed-methods analysis.

First, we should focus on the pathways and barriers to care which relate to healers, especially across Nepal and other LMICs within Asia. While we support structured survey instruments in principle, blindly replicating large-scale, epidemiological studies which cater to Western definitions of psychological illness, wellness, and care may provide misleading, if not harmful, results [1, 69]. Second, we should focus on the mechanisms of healing, both by understanding the healer’s methodology, but also the patient’s subjective experience of that methodology. We have thus far tested our pilot project’s interview protocol to assess the healer landscape and a structured observational rating scale to assess the healer’s commonalities with conventional psychotherapy [54]. Another, though uncharted, avenue would use functional imaging techniques to measure shared brain states between patients of conventional psychotherapy and traditional healers [70].

Lastly, given that the most viable option for collaboration is simply cross-cultural understanding and perhaps early training among biomedical providers, studies that further explore and test collaboration will be critical for the holistic palliation of distress in the context of illness. Although we cannot rule out collaborative models that focus on biomedicine or two-way referral models, it is doubtless cost and resource effective to focus on models which target early education as a way to prevent miscommunication and stigma. Thus, areas for further inquiry include the medical curriculum (medical auxiliary’s curriculum) where students can engage in potentially more discussion about working with traditional healers, understanding traditional healers, and make it less taboo in health worker education. These sessions could also facilitate more interpersonal interaction among traditional healers and health workers.

## Conclusion

Participants from our study reported a nuanced description of what it means to feel mentally well. Some dismissed western knowledge assumptions and circumvented well-funded and replete mental health programs in favor of the healer’s arguably psychotherapeutic treatment. While participants supported collaboration between healers and clinicians, Nepal, like other LMICs, may lack the necessary structural support for more involved models, e.g. training healers on how to task-shift, establishing two way referral systems, as seen within HICs. On the other hand, collaborative education may provide a practical alternative that taxes less resources and effort. Given these findings, healer practices present an opportunity to deliver mental healthcare among the underserved while appealing to indigenous notions of mental well-being. Furthermore, if indigenous practices do hold unique psychotherapeutic value, then we ought to understand that value so as to inform psychotherapy and cross-collaboration as a whole. Overall, studying healers is a matter of urgency given that with the marching advance of western medicine such indigenous therapies may disappear altogether. Thus, further empirical work to study and collaborate with healers is required on a local and a global level while healers still exist.

## Supplementary information

**Additional file 1.** Key illustrative quotes.

**Additional file 2.** A research and policy framework for collaboration between healers and medical providers.

## Data Availability

Data are not publicly available as the information shared in the transcripts make it possible to identify the respondents. As such, this data is available from the corresponding author on reasonable request.
