# Supplementary material for: Traditional and biomedical care pathways for mental well‐being in rural Nepal
Source: Int J Ment Health Syst. 2021 Jan 7;15:4. doi: 10.1186/s13033-020-00433-z (PMC7792081; doi:10.1186/s13033-020-00433-z)
Supplement: Supplementary file 1 — Additional file 1. Key illustrative quotes. [file 13033_2020_433_MOESM1_ESM.docx]

**Appendix S1.** Key Illustrative Quotes

1. “I killed this snake one time. Then later that night I developed a fever because that snake stole my *saato* (spirit). The next day we (her family) called [a *dhami* (healer)] and asked him to perform *phukne* (a traditional treatment of blowing *mantra* on the sick individual), and I got better. So how can people say we (Nepalis) don’t need *dhami* (healers)?” (female homemaker, age 32)
2. “Previously there used to be a large number of people who went to the *dhami-jhankri* (healer). They treated all sorts of medical illnesses and also *bhut pret* (ghost possession) and *hawaa lagne* (supernatural affliction by the wind). These days people still go to the *dhami-jhankri*, but they don't totally depend on them either.” (female homemaker, age 33)
3. “A two-month old once grew sick. The [child’s] father sent the two-month old for *jharfuk* (healer blowing treatment) over and over again. When one *dhami* (healer) failed, he’d just take the child to another, again for simple *jharfuk*. He was poor and just going back and forth to *dhami*. By the time a doctor saw the child they couldn’t do anything so the child died. This happened because in the village we believe more on *dhami* and give priority to them.” (female homemaker, age 68)
4. "The *dhami-jhankri* doesn't demand any money for their services, so when we see the *dhami-jhankri* we don't pay them with money. Generally, out of thanks, we give the *dhami-jhanki* something to eat and drink like tea. It would be good to pay them nicely but at the time we didn't have anything so what to do." (female homemaker, age 36)
5. “My friend asked me to go with her to the *mata* (female healer), because she was too scared to go alone. I heard rumors that the *mata* shivers and vomits in front of her patients. I also heard she predicted two events which both came true. She was too scary.” (female homemaker; age 35)
6. "It's similar to seeing the doctor. You want to go to a doctor who specializes in your problem. In the same way, if you haven't been cured by one *dhami-jhankri* (healer) then you go to another (healer) thinking that the first one did not specialize in your problem." (male mechanic; age 30)
7. “I have to agree to all patient requests. If I refuse then others will say that I was acting *thulo bhayo* (arrogant). I have to go there, see their *jokana* (astrology), perform *jhaarphuk* (treatment of blowing illness away), and tell the patient that they’ll be okay.” (male healer, age 53)
8. “I have an uncle who is Christian. He along with the rest of my family suffered from jaundice. My father and another uncle took the medicines of the *dhami-jhankri* (healer) and got better. On the other hand, my Christian uncle just kept seeing the doctor and never got better. His neck grew thin, and as he lay dying, he would tell us, ‘We should not eat these medicines! We should not use *dhami-jhankri*.’ Then all the villagers including my father recommended that he see the *dhami-jhankri*. He didn’t want to at first, but eventually he went to the *dhami-jhankri* who instructed him to perform *bhakal* (an offering to the deities) using *boka* (a male goat). While he refused to offer a goat he did offer money. And when the *dhami-jhankri* offered that money to the [Hindu] deities my uncle got better." (Female Community Health Volunteer, age 47)
9. "I was having trouble with my pregnancy so I went for a checkup at [location of hospital]. I took their medicine and got better but then later I developed gastric (catch-all term for various ailments related to the gastrointestinal tract) so I went to the healer. Sometimes I went to healers. Sometimes I went to doctors. Sometimes I went to healthposts for their medicines. Sometimes I went to [name of specific healer]." (female farmer, age 36)
10. ”While walking in the streets, you may feel the wind and this may be *garmi bayar* (affected by heat). The *dhami-jhankri* will call this *hawaa lagne* (afflicted by the wind) or even *pret lagcha* (afflicted by ghosts). The doctor can’t help in these cases because he provides the injections. The doctor won’t know whether their patient has *hawaa lagne* or other *lagne* (general term for supernatural afflictions) like that. But you know, the *dhami-jhnakri* (healer) would know and the *dhami-jhankri* would perform *jhaarphuk* (blowing treatment). This is critical, because if a patient has *hawaa lagne* then the doctor’s medications will not work until the *dhami-jhankri* performs their *jhaarphuk*. That’s why a patient who is suffering from both *hawaa lagne* and medical illness must both see a *dhami-jhankri* and take medicine for a quick cure.” (female homemaker, age 70)
11. “If medications do not help us get better then we go to see the *dhami-jhankri* (healer). We mostly go to the *dhami-jhankri* for ourselves -- for our own *atma santushti* (self-satisfaction). We also go just to make sure that we are doing everything we can. When we go to the doctor they'll tell us that we are doing the right thing and that we should keep consuming medicine. However, the doctors won't say to go to the *dhami-jhankri*. Regardless, we will go for our own *atma-santushti*.” (male farmer; age 28)
12. "When people seek treatment for medical illness they do so with an immediate cure in mind. Let’s take you for example. If you get medicines from the doctor for a fever and feel better the next day then you'll feel relieved. You'll feel that the medicine worked. On the other hand, if you didn’t feel better the next day then you'll look for another doctor or another *dhami-jhankri*. It doesn’t matter who." (male farmer, age 43)
13. “In the hospital, if a patient suspects that they might be suffering from *laagnu* they may doubt the doctor's medicines and want to use the *dhami-jhankri* (healer), but the doctors won't allow the *dhami-jhankri* into the hospital to perform their rituals. Instead the *dhami-jhankri* will have to perform their treatments outside the hospital premises. Under these circumstances I've known some patients who have requested their discharge early just to seek thetreatment of the *dhami-jhankri*.” (male mechanic; age 43)
14. "My sister (the niece's mother) said nothing would happen. My niece just drank some water and slept. Then after one or two hours my niece looked pale and my sister started panicking. She called the *dhami-jhankri* who gave my niece some chilis. He calmed my sister but after some time that night my niece died.” (Female Community Health Volunteer, age 47)
15. “In the village patients mostly go to *dhami* (healers) rather than doctors. That’s why the [name redacted] government trained *dhami* and provided them with licenses. At the VDC (Village Development Committee) office, the government officials taught *dhami* how to diagnose certain conditions and when to refer to the hospital. The program would say ‘These are patients that you have to see and these are patients that doctors have to see.’ Since the training, *dhami* now perform their treatment just once and then tell the patient, ‘if you are not feeling better then go to the doctors.’ That was one good part of the training.” (male healer, age 90)
16. “One time, hospital officials told me I had to provide two copies of my photograph. Well, I provided them these items and to this day neither they nor the rural municipality has provided me with documents or proof that I work here at this temple. It has been a year since I submitted my application but they have not given me anything since. Why are they not providing me this? Isn't this discrimination?” (female healer, age 43)
17. “I worked as a *dhami* (healer) from childhood and have cured patients. Other times I could not heal patients which doctors could. We (healers) feel that better coordination between doctors and *dhami* would help us (healers) treat *laagu* (supernatural affliction) and doctors treat medical disease.” (male healer, age 42)
18. “Doctors and *dhami-jhankri* (healers) should consult each other more often. We (healers) have to perform *panchaune* (treating spirit affliction). But if they need to see the doctor then they should see the doctor. Even our sons don't believe in *dhami-jhankri* so when we (the older generation of healers) get sick they take us to the doctors. But then again even the doctor would ask patients such as us (the older generation of healers) if we've seen the healer yet. So, with all that said and done, no one can say only they can cure the patient. We (providers on the whole) have to suggest for patients to go and get the treatment from other providers as well.” (male healer; age 63)
19. "It would work well if *dhami-jhankri* (healers) and doctors could share a space to treat patients. If the doctor would arrive to the patient's home at the same time as me, I would say, 'It's okay. I am also doing my work so you can also do your work.' Whatever we do should be to help the patients with their suffering." (male healer, age 68)
