# Supplementary material for: Traditional and biomedical care pathways for mental well‐being in rural Nepal
Source: Int J Ment Health Syst. 2021 Jan 7;15:4. doi: 10.1186/s13033-020-00433-z (PMC7792081; doi:10.1186/s13033-020-00433-z)
Supplement: Supplementary file 2 — Additional file 2. A research and policy framework for collaboration between healers and medical providers. [file 13033_2020_433_MOESM2_ESM.docx]

**Appendix B. A Research and Policy Framework for Collaboration Between Healers and Medical Providers**

Participants most frequently volunteered three general approaches to collaboration between traditional healers and medical providers. In the first approach, programs would train and supervise healers on how to recognize biomedical issues and when to refer, e.g., potential emergencies. Participants who proposed this idea drew inspiration from personal accounts of when healers in Nepal once “certified” into the ranks of biomedicine. If we examine the history of healthcare in Nepal we can indeed find discussions about such accreditation [1–5]. For instance, since at least the 1970s, Oxfam proposed collaboration in Nepal which would focus on the healer as a “community health leader” [6–8]. Then around 1986, organizations such as the UK Save the Children Fund, United Mission to Nepal, and the Britain Nepal Medical Trust Community Health Development Program took stock of these suggestions and trained healers in western medicine as a means to close the primary health care gap [9,10]. For example, Save the Children alerted healers to nearby healthposts services including family planning, infant immunizations, and scabies treatment, trained healers on how to recognize basic medical issues such diarrhea, early malnutrition, and communicable diseases, and encouraged healers to refer to these health posts using a referral slip program. To combat reversible illnesses, some healers even learned how to fashion home remedies such as nutritious flour, basic first aid, and oral rehydration supplements [11,12].

Continuing upon this trend, the Nepal Netra Jyoti Sangh, an NGO, and the United Nations Development Programme trained healers in basic healthcare subjects related to common medical illnesses, first aid kits, contraception, [13,14], eye care [15,16], and the HIV transmission and prevention [17]. Alternatively, Subba and colleagues [18] took a more mental health focus by training and assessing healers in using the Community Informant Detection Tool (CIDT), a Nepali and community-setting validated instrument for identifying depression, psychosis, alcohol use problems, epilepsy, and child behavioral problems. In view of these efforts, which have spanned nearly five decades, we must consider why evidence of their sustainability has rarely appeared in recent literature such as ours.

First, simple reflection upon findings from this study reveals a number of physicians who discredited the reality of healers operating within the community, let alone as health actors for further collaboration. Other community members deemed biomedicine and traditional healing as too fundamentally different for reconciliation. Within the broader research, Ian Harper (2014) [12], who had previously worked with the aforementioned Save the Children, the United Mission to Nepal Hospital, and the Community Health Development Program, linked the lack of sustainability to several shortcomings. First and foremost, bureaucracy related to these programs could hamper their overall efficiency. Second, broader, simultaneous efforts by the state, for-profit industries, and the media shaped a new biomedical hierarchy in which medical science stood at the top and local health-related theories at the bottom. This hierarchy, rather than integrating, in effect ostracized magico-religious beliefs and practices in the name of progress, science, and development. For example, a state-sanctioned medical order stereotyped the healer as a backwards “imposter” whose only appropriate recourse towards modernization would be persistent biomedical training. The media waged similar battles against healers, labeling them as counter-productive towards development – "This [healer] tradition does nothing except push people toward darkness," [19]. Harper, present at a training for healers led by United Mission to Nepal Hospital and the Community Health Development Program, recalled a general discomfort when even discussing traditional conceptions of illness. Ultimately, the government discontinued the program by Save the Children not to further discredit the healer, but out of fear that they would come to demand due compensation with, rather surprisingly, growing credibility.

In another telling example, Reissland [20], worked on a program which integrated modern and traditional obstetric services in rural Nepal and in the process noted, “On the one hand, medical staff see themselves as a modern elite, restoring health to people who are 'backward', 'superstitious', 'ignorant' and 'destitute'. On the other hand, they perceive that their hospital, underfunded and understaffed, is constrained by the very same forces which impede the advancement of the people whom they are professionally committed to healing. Local, 'non-scientific' health care practices enter the provincial hospital not as part of a formal government plan to integrate traditional healing within the national health system, but as an admission of the hospital's inability to impose the normative form of their organization which obtains at the national capital**.”** More recently, Subba and colleagues [18] discovered other flaws with a biomedically-focused approach. For instance, they discovered barriers among healers themselves, particularly in their ability to comprehend and apply the CIDT when compared to female community health volunteers and mothers’ group members.

This leads us to the second proposal by our participants – traditional and biomedical systems working in compliment with each other through two-way referral pathways. In this manner, healers would continue treating in ways that may have long since improved indigenous psychological wellness while clinicians would continue with evidence-based care. Furthermore, a two-way approach affords a wider epistemological space that would remove the perceived contradictions between traditional and biomedical systems and compel less patients to silo their care in one camp or the other. By connecting the two sides, healers would in effect destigmatize mental illness, increase medical compliance, and dissuade patients from duplicating care among multiple, equally-qualified providers. Lastly, preserving the healer’s role would also, at the behest of some community members, preserve Nepali culture against the growing trend of westernization.

Numerous works from Nepal have acknowledged the healer’s non-biomedical role in treating mental and psychosocial health issues [21], however, to our knowledge a two-way referral system has yet to surface in Nepal. In fact, two-way collaboration has scarcely emerged within LMICs as a whole when compared to HICs [22]. Thus, like the biomedically-focused model, we again face the question of why two-way models lack sustainability, especially within LMICs.

One answer may rest in the most popular proposal among our participants. They encouraged a model of complimentary care sustained not by referring, but by mutual acknowledgment and understanding. In comparison to the two previous approaches, focusing on an educational system would save time, energy, and resources -- factors that could prove critical for sustainability within low- and middle-income countries [22]. For example, healers could teach classes for professional development and doctorate education [23]. Here, facilitating healthy discussions between healers and clinicians would create a collegiate atmosphere and offer opportunities to learn from, check, but not to subsume the other. However, to do so such programs would have to promote not only “cultural competency” among its biomedical providers, but also treatments for the community by the community, otherwise referred to as “culturally commensurate” therapies [24]. By respecting the epistemological space of another, providers would align with, rather than against pre-existing pathways to healer care, again compelling less patients to silo or hide their care pathways. This would also avoid a check-list-based approach that would reduce the essence of culture and promote a false sense of security among biomedical providers.

In Nepal, Shankar [25] has fittingly encouraged a medical school curriculum that stressed the role of integrative medicine and healers. To this end, he spotlighted one example in which Kathmandu University launched a pilot course on integrative medicine for its medical students. While medical students from this course expressed an overall favorable attitude towards learning about integrative medicine, we should also emphasize their reluctance to implement healers in conjunction with allopathic treatments [26,27]. Despite these shortcomings, education about and understanding traditional healing may provide the most feasible pathway towards culturally commensurate therapies among underserved rural populations in low- and middle-income countries such as Nepal.

**References**

1. Hoff W. Traditional healers and community health. InWorld Health Forum. 1992;13:182–7.

2. Oswald IH. Are traditional healers the solution to the failures of primary health care in rural Nepal? Soc Sci Med. 1983;17:255–7.

3. Dhakal R, Graham-Jones S, Lockett G. Traditional Healers and Primary Health Care in Nepal. Kathmandu, Nepal: Save the Children Fund – United Kingdom; 1986.

4. Gillam S. The traditional healer as village health worker. J Inst Med. 1989;11:67–76.

5. Shrestha R, Lediard M. Faith healers, a force for change: Preliminary report of an action-research project. Nepal: Kathmandu: UNFPA; 1980.

6. Eade D, Williams S, Oxfam. The Oxfam handbook of development and relief. Oxford: Oxfam; 1995.

7. Subedi J. Primary Health Care and Medical Pluralism Exemplified in Nepal: A Proposal for Maximizing Health Care Benefit. Sociol Focus. Routledge; 1992;25:321–8.

8. Sharma V. Prevalent opinion of traditional healers about clients who faint or are possessed. Int Workshop Epilepsy Conduct Jointly EPICADEC Dep Psychiatry IOM TUTH. Kathmandu, Nepal; 1996.

9. Gibbon M, Cazottes I. The health analysis cycle. Nepal: Britain Nepal Medical Trust; 1997.

10. Pigg SL. Acronyms and effacement: traditional medical practitioners (TMP) in international health development. Soc Sci Med 1982. 1995;41:47–68.

11. Sharma A, Ross J. Nepal: integrating traditional and modern health services in the remote area of Bashkharka. Int J Nurs Stud. 1990;27:343–53.

12. Harper I. Development and Public Health in the Himalaya: Reflections on healing in contemporary Nepal. 1 edition. London ; New York, NY: Routledge; 2014.

13. School and Community Health Project. A Traditional Healers Training Manual of School and Community Health Project. Kathmandu, Nepal; 1996.

14. Poudyal AK, Jimba M, Murakami I, Silwal RC, Wakai S, Kuratsuji T. A traditional healers’ training model in rural Nepal: strengthening their roles in community health. Trop Med Int Health. 2003;8:956–60.

15. Poudyal B. Traditional healers as eye team members in Nepal. Community Eye Health J. 1997;10:4–5.

16. Poudyal AK, Jimba M, Poudyal BK, Wakai S. Traditional healers’ roles on eye care services in Nepal. Br J Ophthalmol. 2005;89:1250–3.

17. Poudel KC, Jimba M, Joshi AB, Poudel‐Tandukar K, Sharma M, Wakai S. Retention and effectiveness of HIV/AIDS training of traditional healers in far western Nepal. Trop Med Int Health. 2005;10:640–6.

18. Subba P, Luitel NP, Kohrt BA, Jordans MJD. Improving detection of mental health problems in community settings in Nepal: development and pilot testing of the community informant detection tool. Confl Health. 2017;11:28.

19. Nepal. Dhami-jhankri: A Mystery (Dhami-jhankri: Ek rahasya). Varanasi Kalpana Press; 1994.

20. Reissland N, Burghart R. Active patients: The integration of modern and traditional obstetric practices in Nepal. Soc Sci Med. 1989;29:43–52.

21. Pham TV, Kaiser BN, Koirala R, Maharjan SM, Upadhaya N, Franz L, et al. Traditional Healers and Mental Health in Nepal: A Scoping Review. Cult Med Psychiatry [Internet]. 2020 [cited 2020 Sep 1]; Available from: https://doi.org/10.1007/s11013-020-09676-4

22. Pham TV, Koirala R, Wainberg M, Kohrt BA. Reassessing the Mental Health Treatment Gap with Traditional Healers. J Community Ment Health. 2020;1–15.

23. Pouchly CA. A narrative review: arguments for a collaborative approach in mental health between traditional healers and clinicians regarding spiritual beliefs. Ment Health Relig Cult. Routledge; 2012;15:65–85.

24. Wendt DC, Gone JP. Rethinking cultural competence: Insights from indigenous community treatment settings. Transcult Psychiatry. SAGE Publications Ltd; 2012;49:206–22.

25. Shankar PR, Paudel R, Giri B, Shankar R. Healing traditions in Nepal. J Am Assoc Integr Med. 2006;

26. Shankar PR, Das B, Partha P, Shenoy N. Medical students’ opinions about complementary and alternative medicine: a questionnaire based survey. J Inst Med. 2003;25:9–12.

27. Shankar P. Student attitude towards integrative medicine in a medical college in Western Nepal: a questionnaire-based study. J Am Assoc Integr Med. 2006;11.
